# We all have “skin in the game” in mental health research: Inaugural editorial

**DOI:** 10.1038/s44184-022-00003-w

**Published:** 2022-05-25

**Authors:** Jack Tsai

**Affiliations:** 1 School of Public Health, University of Texas Health Science Center at Houston, Houston, TX USA; 2 Department of Psychiatry, Yale University School of Medicine, New Haven, CT USA; 3 National Center on Homelessness among Veterans, U.S. Department of Veterans Affairs, Tampa, FL USA

It is my honor to serve as the inaugural Editor-in-Chief and to write the first editorial for *npj Mental Health Research*. With an esteemed team of Associate Editors, and leadership staff at Springer Nature, *npj Mental Health Research* aspires to be an international public platform for researchers, providers, patients, and policymakers to publish and share their work on mental health with the world.

We are living in both frightening and exciting times. With new emerging problems, there are opportunities for research and innovation. Epidemics, wars and human conflicts, changing climates, and technological advancements are changing the way we live, work, and play. Science is trying to keep up, but it’s hard and our understanding of human behavior and mental health is still in nascent stages. There is now more information available at our fingertips than ever before in history, yet there is still so much we do not know about ourselves. At a micro level, each of us are affected by mental health in our lives and nearly all of us likely know somebody that struggles with mental health. At a macro level, the productivity and functioning of societies depends on the mental health of its citizens. The important scientific frontier of mental health may be essential to our well-being and survival as the world around us evolves.

With that context, allow me to take this opportunity to elaborate on the aims of *npj Mental Health Research*. The first aim is to publish scientific work on novel approaches to the conceptualization, assessment, and treatment of mental health and substance use disorders. There is a high and increasing rate of mental health and substance use problems in many countries that would benefit from improved and innovative ways to identify, engage, and treat these problems. The second aim is to foster interdisciplinary and collaborative research with diverse experts including unique private-public partnerships. The field of mental health is increasingly becoming interdisciplinary because we need experts from diverse fields to bring their expertize, methodologies, and technologies to mental health research. The third aim is to highlight studies on new technologies for mental health research and social determinants of mental health. The 21st century has brought many new technologies that have changed the way we live, work, and play; and there are so many new technologies on the horizon that will transform human living and human behavior.

Together, with these three aims of the Journal, we plan to feature topical and timely articles to inform the mental health field and the broader public. We understand the need to engage stakeholders in different ways so we will work to have a social media presence to highlight certain articles and develop new engagement and dissemination methods. We look forward to reaching diverse audiences and invite you to participate in this development.

To ensure universal access to our content, *npj Mental Health Research* is an open-access journal which means articles in the Journal will be freely accessible to anyone free-of-charge. To make this sustainable, authors who are submitting their work will be asked to pay a publication fee that hopefully their institutions can support.

In conclusion, we welcome your involvement in the Journal. We will be looking for contributors to the science of mental health. We welcome Research Articles, as well as Comments and Perspective papers. We greatly value Review papers, whether they are systematic review or meta-analyses so we will particularly appreciate those type of submissions. In addition to submitting your work, we also welcome you to review the work of others. We are always looking for astute Reviewers to review the manuscripts we receive and are grateful for them to offer their expertize. Peer review is the gold standard of scientific evaluation and it relies on the often-thankless work of Reviewers but we are very grateful to them. When it comes to mental health research, we all have “skin in the game” to help optimize mental health, maximize human potential, and encourage human thriving and flourishing.

